# Clinical value of biomechanics and magnetic resonance imaging in the evaluation of knee osteoarthritis

**DOI:** 10.3389/fsurg.2026.1751286

**Published:** 2026-03-09

**Authors:** Jun Zhou, Cheng Guo, Guifang Liu, Yaofei Liu

**Affiliations:** 1 Department of Medical Imaging, Affiliated Hospital of Traditional Chinese Medicine of Xinjiang Medical University, Urumqi, Xinjiang Uygur Autonomous Region, China; 2 Department of Molecular Imaging, Qingdao Central Hospital, Qingdao, Shandong, China; 3 Department of Radiology, Qingdao Central Hospital, Qingdao, Shandong, China; 4 Department of Medical Imaging, Xi'an NO.5 Hospital, Xi'an, Shaanxi, China

**Keywords:** biomechanics, clinical value, diagnostic efficacy, knee joint, magnetic resonance imaging, osteoarthritis

## Abstract

**Objective:**

The aim of this study was to analyze the clinical value of biomechanics and magnetic resonance imaging (MRI) in the evaluation of knee osteoarthritis.

**Methods:**

Sixty patients diagnosed with knee osteoarthritis from June 2020 to November 2023 in our hospital were retrospectively selected as the study group. Fifty healthy subjects who underwent annual health checkups in our hospital during the same period were selected as the control group. A three-dimensional finite element model was constructed based on knee MRI images. The MRI parameters and biomechanical parameters of knee joints were compared between the two groups.

**Results:**

The mean Whole-Organ Magnetic Resonance Imaging Score (WORMS) of patients in the study group was (72.29 ± 16.92), significantly higher than that of (44.68 ± 16.95) in the control group. The contact area between the medial femoral cartilage and medial meniscus, the maximal von Mises stress on the medial meniscus, and the maximal von Mises stress on the femoral cartilage were significantly greater in the study group than in the control group. The area under the curves of MRI indicators and biomechanical indicators (contact area between the medial femoral cartilage and medial meniscus, the maximal von Mises stress on the medial meniscus, and the maximal von Mises stress on the femoral cartilage) for knee osteoarthritis were 0.8694, 0.7874, 0.6282, and 0.7650, respectively.

**Conclusion:**

WORMS and biomechanical parameters (medial femoral-meniscal contact area and peak stress) demonstrate good diagnostic value in knee osteoarthritis, with the maximal von Mises stress on the medial meniscus showing discriminatory power for disease severity. The combination of MRI and biomechanical analysis facilitates the assessment of knee osteoarthritis from both structural and functional perspectives, providing objective evidence for clinical diagnosis and treatment.

## Introduction

Osteoarthritis, also known as degenerative arthritis, is a type of chronic joint disease and a common cause of joint pain, deformity and even limb disability (1). The knee joint is the most crucial load-bearing and locomotor joint in the human body, characterized by an extremely complex structure. Given that it bears the main load of the body and is subjected to significant activity, it has the highest injury rate among all joints, making it one of the most common sites for osteoarthritis (2). The typical pathological features of knee osteoarthritis include degeneration of articular cartilage and synovitis. Additionally, most patients experience joint pain and functional impairment, and in severe cases, total joint replacement may be necessary (3, 4). Practice has revealed that knee osteoarthritis is the most prevalent joint disease among the elderly, and in people over 50, it ranks second only to cardiovascular diseases in causing long-term disability, significantly impacting patients' health and quality of life while increasing socioeconomic burdens; data indicate that knee osteoarthritis has become one of the leading causes of disability worldwide (1, 5).

Knee osteoarthritis can be attributed to the degeneration of joint cartilage. An epidemiological study (6) has confirmed that the severity of cartilage degeneration in patients with knee osteoarthritis is generally positively correlated with the severity of their clinical symptoms. Etiological research has indicated that the pathogenesis of knee osteoarthritis results from the combined effects of biological and biomechanical factors, leading to an imbalance in the synthesis and degradation processes of chondrocytes, extracellular matrix, and subchondral bone, ultimately triggering knee osteoarthritis (7). The biomechanical behavior of knee osteoarthritis is highly complex. The integrity of the knee joint structure is crucial for maintaining the stability of the internal biomechanical environment, whereas damage to the knee cartilage, meniscal injuries, and surgical procedures can all lead to biomechanical changes in the knee joint (8). Magnetic resonance imaging (MRI) technology, renowned for its radiation-free nature and high resolution, is extensively utilized in clinical medical examinations. Existing studies (9, 10) have confirmed its significant value in assessing cartilage wear in the knee joint. This study aimed to retrospectively analyze the clinical value of applying MRI for biomechanical analysis in patients with knee osteoarthritis and preliminarily assess the diagnostic efficacy of quantitative MRI and biomechanical indicators for knee osteoarthritis.

## Materials and methods

### Study design and patient inclusion

This study was approved by ethics committee of the Xi'an NO.5 Hospital. All patients or their families provided written informed consent. A retrospective cohort study design was used. Utilizing the hospital's electronic medical record system, 60 patients diagnosed with knee osteoarthritis in our hospital from June 2020 to November 2023 were selected as the study group based on the inclusion and exclusion criteria, while 50 healthy individuals undergoing medical checkups during the same period were chosen as the control group. The control group consisted of individuals undergoing routine annual health checkups at our hospital. These individuals underwent routine physical examinations for occupational or personal health management purposes. None of them complained of knee-related symptoms. After clinical assessments and imaging examinations, knee osteoarthritis and other knee joint diseases were ruled out.

Inclusion criteria: (1) patients in the study group met the diagnostic criteria for knee osteoarthritis established by the American College of Rheumatology (ACR) (11). The ACR criteria were used as the inclusion criteria because they integrate clinical symptoms, signs, and imaging manifestations, which facilitates applicability in clinical practice. The KL classification was used to stratify disease severity, facilitating subsequent subgroup analysis; (2) both the study and control groups underwent knee joint MRI examinations and biomechanical analyses; (3) baseline clinical data such as gender, age, and body mass index (BMI), MRI indicators, and biomechanical indicators (contact area between the medial femoral cartilage and medial meniscus, the maximal von Mises stress on the medial meniscus, and the maximal von Mises stress on the femoral cartilage) were complete for both the study and control groups; (4) all patients in the study group had unilateral lesions.

Exclusion criteria: (1) patients with concurrent psychiatric disorders; (2) patients with systemic immune system diseases; (3) patients with chronic infections; (4) patients with severe liver or kidney dysfunction; (5) pregnant or breastfeeding women; (6) individuals dependent on alcohol or drugs.

Based on the above inclusion and exclusion criteria, a total of 60 patients with knee osteoarthritis were included in the study group, and 50 healthy individuals were included in the control group. Disease severity was further stratified according to the Kellgren-Lawrence (KL) classification. Among the 60 included patients, 12 (20.0%) were classified as KL grade I, 23 (38.3%) as grade II, 18 (30.0%) as grade III, and 7 (11.7%) as grade IV. Patients in the study group were stratified into a mild group (KL grades I − II, *n* = 35) and a severe group (KL grades III − IV, *n* = 25) according to disease severity to analyze the differences in various indicators between patients with different disease severity.

### Data collection

The baseline clinical data, including gender, age, BMI in the study and control groups, and disease duration in the study group, were collected and analyzed. The quantitative knee MRI indicators of Whole-Organ Magnetic Resonance Imaging Score (WORMS) were gathered for both groups utilizing the Umr 780 3.0T MRI scanner. Patients were placed in a supine position with knees extended. The scanning sequences included spin-echo T1W1 and gradient-echo T2W1. The WORMS scoring system (12) was used to divide the knee into four parts: the medial tibiofemoral joint, the lateral tibiofemoral joint, the patellofemoral joint, and intercondylar eminence, including a total of 15 regions, evaluating cartilage signal morphology, articular surface, subchondral cysts, and subchondral bone attrition, with higher scores indicating more severe knee structural damage. The WORMS assessment was independently performed by two radiologists with more than 5 years of experience in MRI interpretation of knee osteoarthritis. Both radiologists received standardized training on the WORMS scoring system before assessment. The intraclass correlation coefficient (ICC) was used to assess interobserver agreement, with an ICC of 0.89 (95% CI: 0.84–0.93), indicating good interobserver agreement. When the score difference between the two assessors exceeded 10%, arbitration was performed by a third senior radiologist. The mean of the three assessments was taken as the final result.

### Biomechanical analysis methods

Since knee osteoarthritis primarily affects the medial compartment (approximately 70%−80% of cases), this study focused on biomechanical parameters of the medial compartment. Finite element analysis was performed to obtain biomechanical parameters, including contact area between the medial femoral cartilage and medial meniscus, the maximal von Mises stress on the medial meniscus, and the maximal von Mises stress on the femoral cartilage. (1) Three-dimensional model construction: Based on knee MRI images (slice thickness: 1 mm; sagittal proton density-weighted images), image segmentation was performed using Mimics 21.0 (Materialise, Belgium) to extract the three-dimensional geometric morphology of the distal femur, proximal tibia, meniscus, and articular cartilage, which were then exported in STL format. (2) The finite element model construction: The three-dimensional geometric models were imported into ANSYS Workbench 2021 R1 software for meshing and finite element analysis. Tetrahedral elements with a mesh size of 1.0 mm were used for the cartilage and meniscus. Material properties (13): Articular cartilage was modeled as an isotropic linear elastic material (elastic modulus E = 15 MPa, Poisson's ratio = 0.45). The meniscus was modeled as a transversely isotropic material (longitudinal elastic modulus E1 = 150 MPa, transverse elastic modulus E2 = 20 MPa, Poisson's ratio = 0.3). (3) Boundary and loading conditions: The knee joint loading condition at the first peak of the stance phase during the gait cycle (approximately 15% of the gait cycle) was simulated. The first peak of the stance phase was selected because it represents the maximum load during the gait cycle, reflecting the maximum stress state of the knee joint during daily activities. The distal tibia was fixed, and an axial compressive load (2.5 times body weight, approximately 1750 N) was applied to the distal femur. Frictional contact was defined between the femoral cartilage and the meniscus, as well as between the meniscus and the tibial cartilage, with a friction coefficient of 0.02. All contact interfaces were modeled as surface-to-surface contact. (4) Result extraction: After completion of calculations, the contact area between the medial femoral cartilage and medial meniscus (mm^2^), the maximal von Mises stress on the medial meniscus (MPa), and the maximal von Mises stress on the femoral cartilage (MPa) were extracted as biomechanical indicators.

### Quality control

To avoid the high error rate associated with single-person data entry, data collection and entry in this study were conducted jointly by two individuals, who supervised each other and verified the data to ensure accuracy.

### Outcome measures and statistical analysis

This study and its outcomes reveal significant differences in quantitative MRI indicators and biomechanical indicators between patients with knee osteoarthritis and healthy individuals undergoing medical checkups. Both quantitative MRI indicator (WORMS) and biomechanical indicators can be used for the diagnosis of knee osteoarthritis.

Data were entered using EXCEL 2021 and statistical analysis was performed using Statistical Package for the Social Sciences (SPSS) 26.0. Normality analysis indicated that all continuous data followed a normal distribution, expressed as (mean ± standard deviation), and intergroup comparisons were conducted using the *t*-test. Categorical data were expressed as rate, and intergroup comparisons were conducted using the chi-square test. The diagnostic efficacy of WORMS and biomechanical indicators for knee osteoarthritis was evaluated by plotting receiver operating characteristic (ROC) curves. The cutoff values were determined by the maximum Youden index (sensitivity + specificity−1). *P* < 0.05 was considered statistically significant.

## Results

### Comparison of baseline clinical data between the study and control groups

The baseline clinical data of patients in the study and control groups, such as gender, age, and BMI, were included for intergroup comparison. The results indicated that there were no statistically significant differences between the two groups in these data (*P* > 0.05), suggesting good comparability (Table 1).

**Table 1:** Comparison of baseline clinical data between the study and control groups ($\bar{x}\pm s$)/(*n* [%]).

General clinical data		Study group (*n* = 60)	Control group (*n* = 49)	*t/χ²*	*P*
Gender	Male	32	29	1.924	0.165
	Female	28	20		
Mean age (years)		56.36 ± 10.23	57.88 ± 12.51	0.777	0.439
Mean BMI (kg/m^2^)		22.51 ± 2.12	21.98 ± 2.65	1.294	0.198
Mean duration of disease (months)		6.35 ± 1.53	-	-	

BMI, body mass index.

### Comparison of quantitative MRI indicators of WORMS between the study and control groups

**Figure 1:**
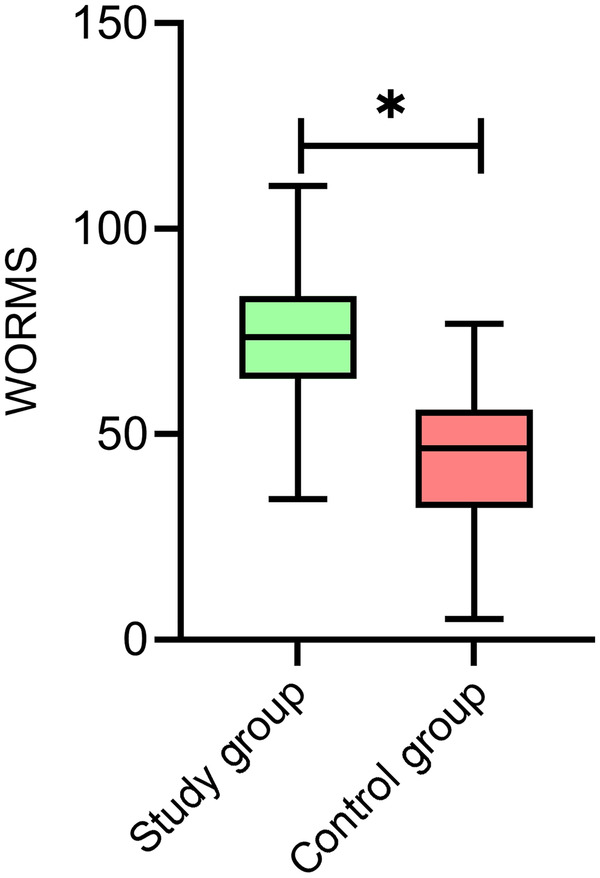
Comparison of quantitative MRI indicators of WORMS between the study and control groups. The mean WORMS of the patients in the study group was significantly higher than that in the control group (*P* < 0.05). * indicates statistically significant difference. MRI, magnetic resonance imaging; WORMS, whole-organ magnetic resonance imaging score.

The WORMS was evaluated using MRI in both groups of patients. The mean WORMS of patients in the study group was (72.29 ± 16.92), significantly higher than that of (44.68 ± 16.95) in the control group (*P* < 0.05) (Figure 1). To better illustrate the characteristics of WORMS in the control group, the scores for each WORMS subregion are presented as follows: cartilage signal abnormalities (12.35 ± 5.21), subarticular cysts (8.42 ± 3.67), bone marrow edema (6.28 ± 2.89), ligaments (5.12 ± 2.34), and others (12.51 ± 4.84).

### Comparison of biomechanical parameters between the study and control groups

**Figure 2:**
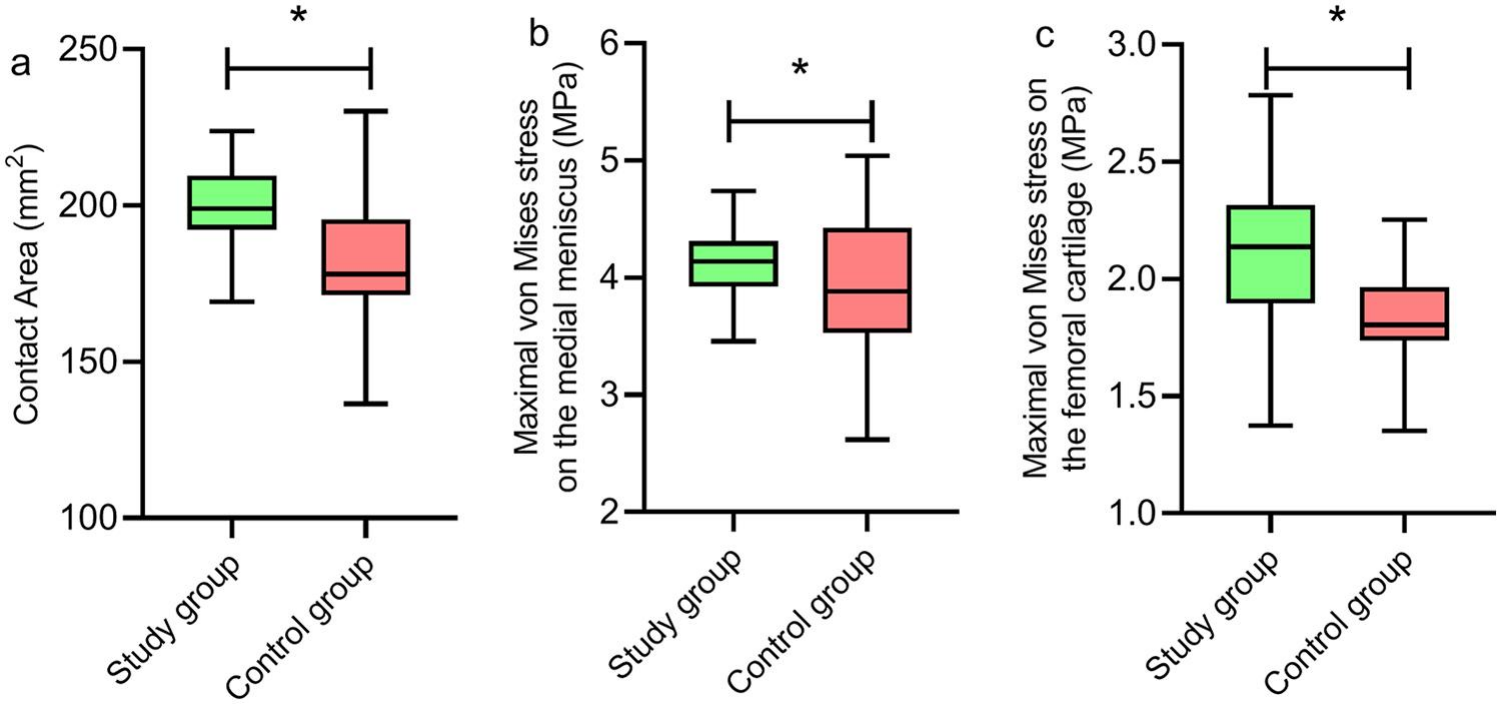
Comparison of biomechanical parameters between the study and control groups. The contact area between the medial femoral cartilage and medial meniscus **(a)**, the maximal von Mises stress on the medial meniscus **(b)**, and the maximal von Mises stress on the femoral cartilage **(c)** in the study group were significantly higher than those in the control group (*P* < 0.05). *P < 0.05.

The contact area between the medial femoral cartilage and medial meniscus in the study group was (200.73 ± 12.03) mm^2^, significantly greater than that of (181.83 ± 21.04) mm^2^ in the control group, with a statistically significant difference (*P* < 0.05) (Figure 2a). The maximal von Mises stress on the medial meniscus in the study group was (4.14 ± 0.29) MPa, significantly higher than that of (3.95 ± 0.56) MPa in the control group, with a statistically significant difference (*P* < 0.05) (Figure 2b). The maximal von Mises stress on the femoral cartilage in the study group was (2.11 ± 0.30) MPa, significantly higher than that of (1.84 ± 0.19) MPa in the control group, with a statistically significant difference (*P* < 0.05) (Figure 2c).

### Analysis of the diagnostic value of MRI and biomechanical indicators for knee osteoarthritis

**Figure 3:**
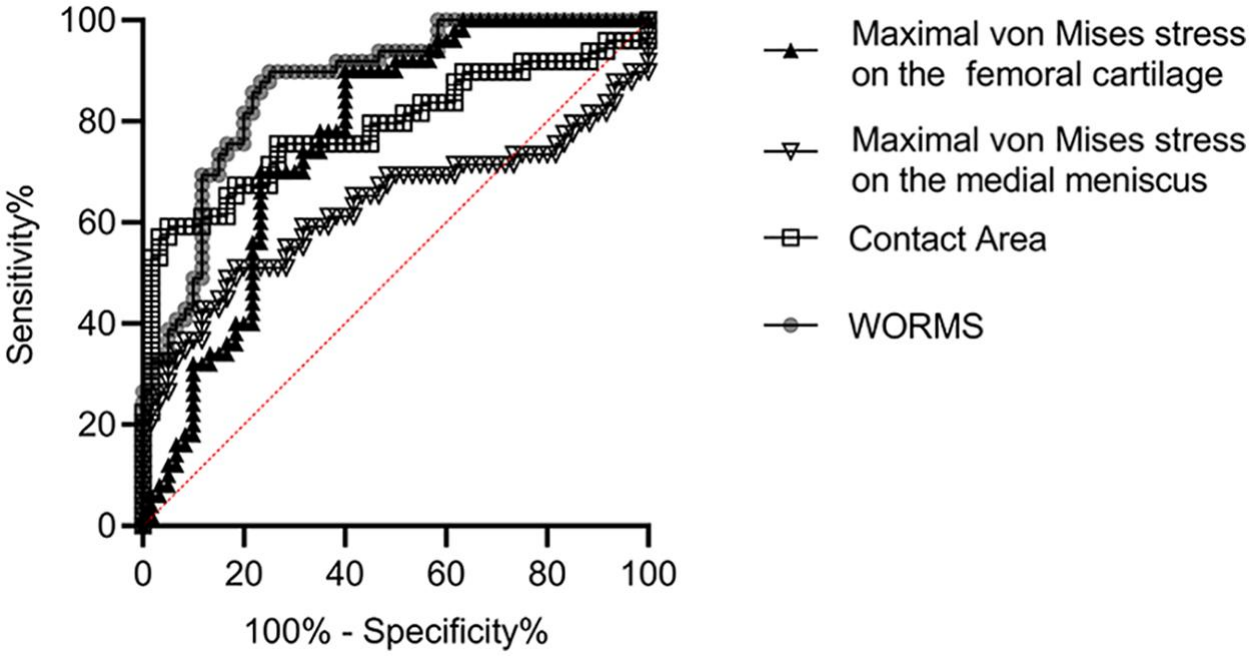
Analysis of the diagnostic value of MRI and biomechanical indicators for knee osteoarthritis. The AUCs of MRI indicators and biomechanical indicators (contact area between the medial femoral cartilage and medial meniscus, the maximal von Mises stress on the medial meniscus, and the maximal von Mises stress on the femoral cartilage) for knee osteoarthritis were 0.8694, 0.7874, 0.6282, and 0.7650, respectively (*P* < 0.05). AUC, area under the curve; MRI, magnetic resonance imaging.

Using ROC curve plotting, the diagnostic value of MRI indicators and biomechanical indicators (contact area between the medial femoral cartilage and medial meniscus, the maximal von Mises stress on the medial meniscus, and the maximal von Mises stress on the femoral cartilage) for knee osteoarthritis were analyzed. The results showed that the diagnostic area under the curves (AUCs) were 0.8694 (95% CI = 0.8033–0.9355), 0.7874 (95% CI = 0.6951–0.8797), 0.6282 (95% CI = 0.5142–0.7423), and 0.7650 (95% CI = 0.6760–0.8540) respectively (Table 2, Figure 3).

**Table 2:** Analysis of the diagnostic value of MRI and biomechanical indicators for knee osteoarthritis.

Diagnostic value	AUC	SE	95% CI	*P*	Cut-off value
WORMS	0.8694	0.0337	0.8033–0.9355	<0.0001	57.64
Contact area between the medial femoral cartilage and medial meniscus	0.7874	0.0471	0.6951–0.8797	<0.0001	192.90 mm^2^
Maximal von Mises stress on the medial meniscus	0.6282	0.0582	0.5142–0.7423	0.0216	4.05 MPa
Maximal von Mises stress on the femoral cartilage	0.7650	0.0454	0.6760–0.8540	<0.0001	1.88 MPa

AUC, area under the curve; SE, standard error; 95% CI, 95% confidence interval.

### Comparison of WORMS between patients with mild and severe knee osteoarthritis

**Figure 4:**
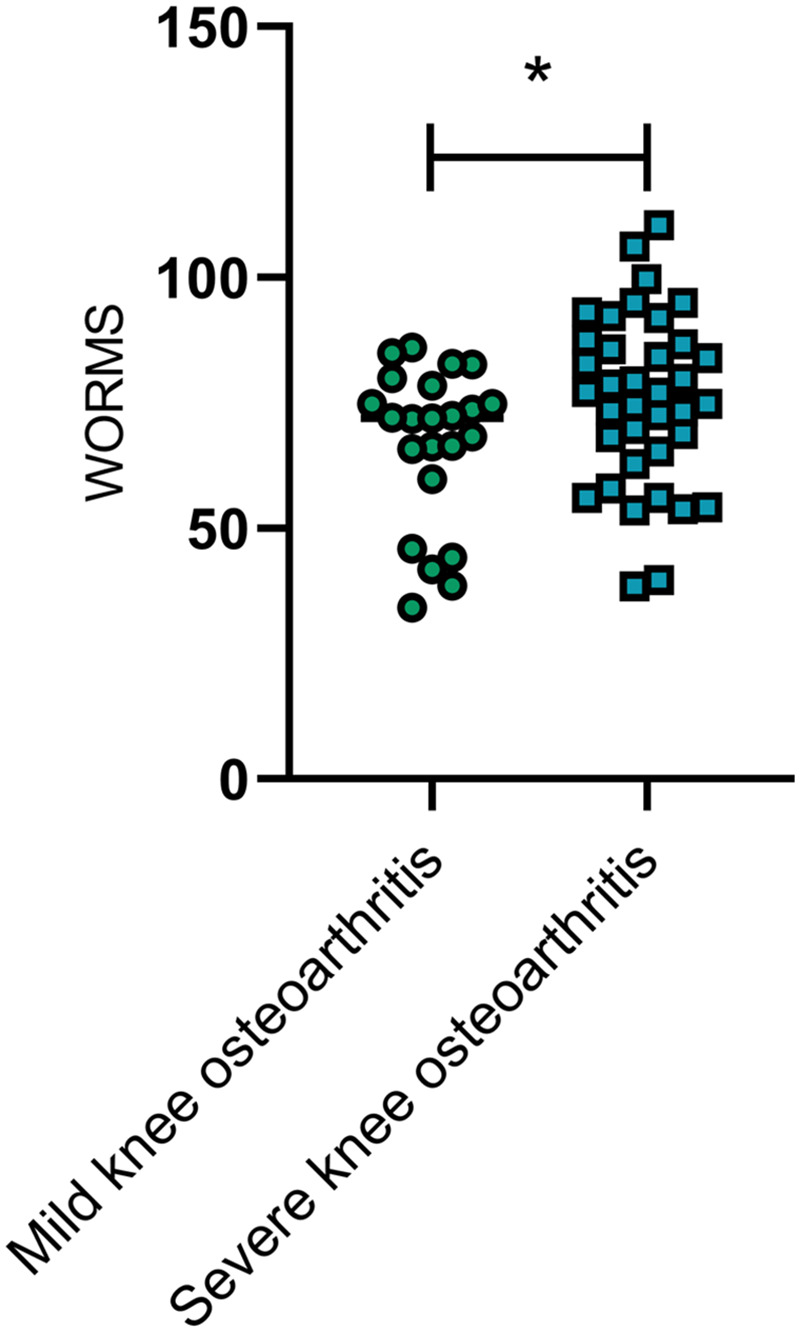
Comparison of WORMS between patients with mild and severe knee osteoarthritis. WORMS was significantly lower in patients with mild knee osteoarthritis than in those with severe knee osteoarthritis (*P* < 0.05). *represents a statistically significant difference between the two groups. WORMS, whole-organ magnetic resonance imaging score.

Patients with mild knee osteoarthritis (KL grade I − II, *n* = 35) had significantly lower WORMS than those with severe knee osteoarthritis (KL grade III − IV, *n* = 25), with a statistically significant difference (*P* < 0.05) (Figure 4).

### Comparison of biomechanical indicators between patients with mild and severe knee osteoarthritis

**Figure 5:**
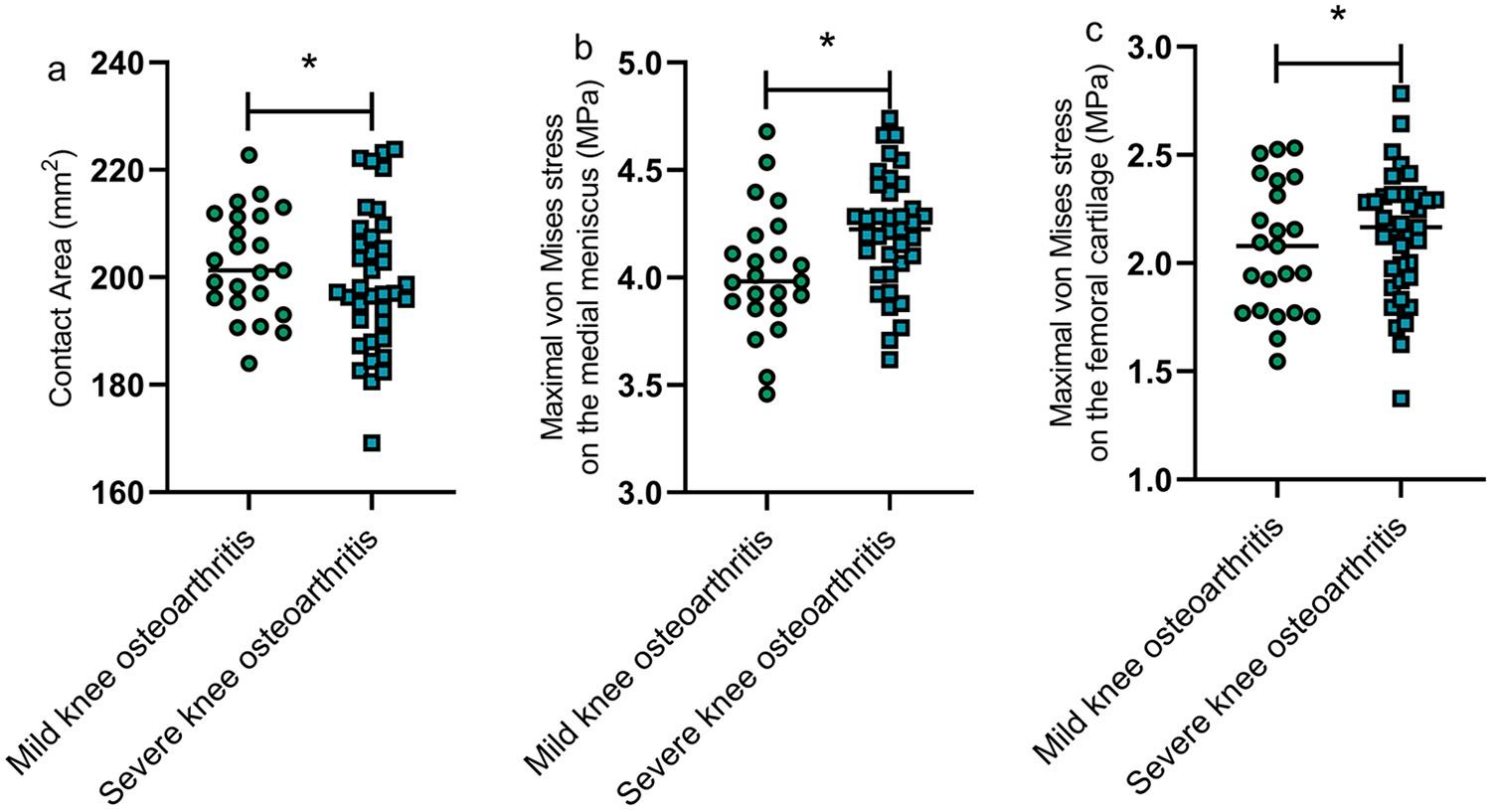
Comparison of biomechanical indicators between patients with mild and severe knee osteoarthritis. The contact area between the medial femoral cartilage and medial meniscus **(a)**, the maximal von Mises stress on the medial meniscus **(b)**, and the maximal von Mises stress on the femoral cartilage **(c)** in patients with mild knee osteoarthritis were significantly lower than those in patients with severe knee osteoarthritis (*P* < 0.05). *represents a statistically significant difference between the two groups.

The contact area between the medial femoral cartilage and medial meniscus (Figure 5a), the maximal von Mises stress on the medial meniscus (Figure 5b), and the maximal von Mises stress on the femoral cartilage (Figure 5c) in patients with mild knee osteoarthritis were significantly lower than those in patients with severe knee osteoarthritis, with statistically significant differences (*P* < 0.05) (Figure 5).

### Analysis of the diagnostic value of MRI and biomechanical indicators for knee osteoarthritis of varying severity

**Figure 6:**
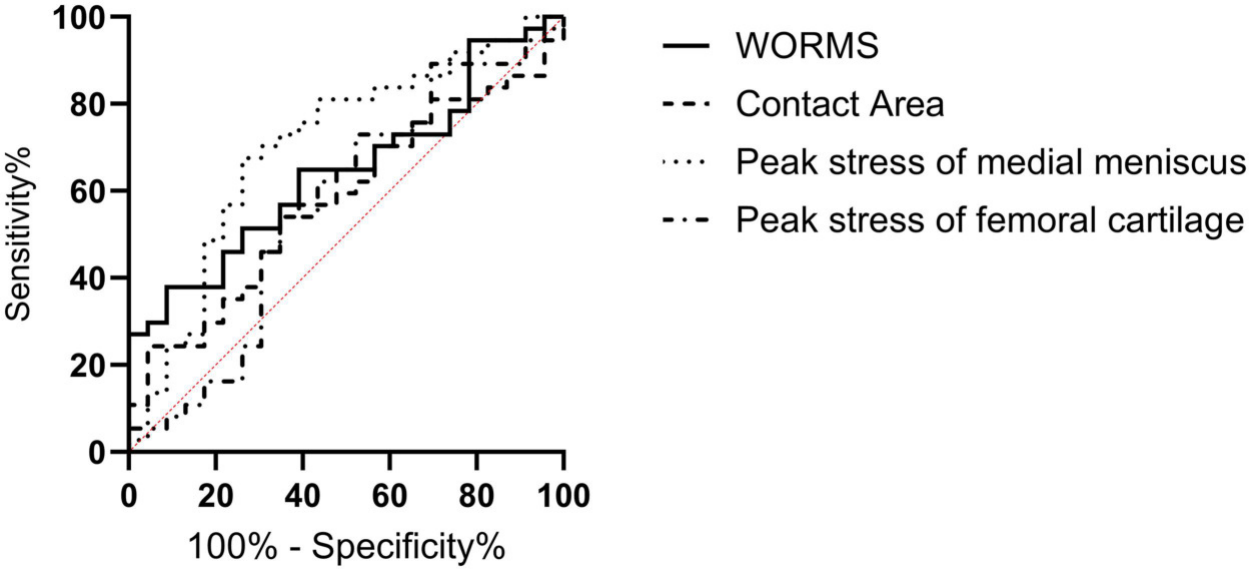
Analysis of the diagnostic value of MRI and biomechanical indicators for knee osteoarthritis of varying severity. The maximal von Mises stress on the medial meniscus had diagnostic value in patients with different severities of knee osteoarthritis, with an AUC of 0.7027 (95% CI = 0.5622–0.8432, *P* = 0.009). AUC, area under the curve; MRI, magnetic resonance imaging; 95% CI: 95% confidence interval.

The ROC curve analysis revealed that only the maximal von Mises stress on the medial meniscus had diagnostic value in patients with different severities of knee osteoarthritis, with an AUC of 0.7027 (95% CI = 0.5622–0.8432, *P* = 0.009), a cutoff value of 4.18 MPa, a sensitivity of 68.0%, and a specificity of 71.4%. Other indicators did not have diagnostic value (*P* > 0.05), as shown in Figure 6.

## Discussion

Epidemiological studies reveal that knee osteoarthritis is one of the common types of arthritis, with a very high incidence rate, particularly among middle-aged and elderly individuals. Data indicate that approximately 10% of men and 18% of women worldwide suffer from knee osteoarthritis; the overall prevalence in China has reached 8.1%, and with the continuing trend of an aging society, the incidence rate is steadily increasing (14, 15). The pathogenesis of knee osteoarthritis remains unclear; however, most studies suggest that genetic factors, environmental influences, age, and obesity all play significant roles in its development and progression. Currently, there is a lack of specific drugs for the treatment of knee osteoarthritis in clinical practice. Treatment primarily focuses on alleviating pain and slowing disease progression, but clinical outcomes are mixed (16). Western medicine advocates the use of non-steroidal anti-inflammatory drugs and glucosamine hydrochloride capsules. If necessary, intra-articular hyaluronic acid injections can be administered. For severe cases, surgical treatments such as high tibial osteotomy or intra-articular debridement are recommended (17, 18).

The implementation of clinical treatments depends on accurate and timely diagnoses. Currently, the diagnosis and assessment of knee osteoarthritis in clinical practice mainly rely on plain radiographs, clinical manifestations, and laboratory tests. Plain radiographs are the preferred method for clinical assessment of osteoarthritis due to their cost-effectiveness, convenience, and good reproducibility. The Kellgren-Lawrence classification is also mainly based on radiographic findings. However, plain radiographs have low sensitivity for early cartilage lesions, meniscal injuries, and synovial inflammation. MRI, owing to its high soft tissue resolution images without the use of ionizing radiation, has unique value for detailed assessment of joint structural integrity. However, but due to its high examination cost, MRI is not routinely used for follow-up monitoring (19). Numerous studies (20) have confirmed the diagnostic efficacy of MRI for conditions such as shoulder labrum injuries, hip joint lesions, and meniscal injuries. However, some clinical practices (21) have pointed out that MRI still has the defects of inability to monitor the dynamic bone and soft tissue movement, and poor effect on bone changes, which need to be supplemented by other diagnostic and differential indicators. This study analyzed the diagnostic efficacy of MRI and biomechanical indicators in knee osteoarthritis using a controlled group design. The results indicated that in terms of the quantitative MRI indicator WORMS, patients with knee osteoarthritis had significantly higher WORMS. The relatively high WORMS in the control group may be because most of the included patients were middle-aged or elderly individuals whose joints had undergone a certain degree of degenerative changes. The WORMS scoring system is particularly sensitive to subtle changes in joint structure. The WORMS is a semi-quantitative, multi-feature, whole-organ evaluation system used to assess the degree of structural integrity of knee osteoarthritis based on knee joint MRI images. A study conducted on 213 patients with knee osteoarthritis (22) found that there was a positive correlation between the severity of knee osteoarthritis in the enrolled patients and their WORMS, and that it was of great significance to apply the WORMS to the assessment of the condition of patients with knee osteoarthritis, which was similar to the results of the present study, suggesting that the WORMS, a quantitative indicator of MRI, is associated with the condition of knee osteoarthritis. In terms of biomechanical indicators, this study found that the contact area between the medial femoral cartilage and medial meniscus, the maximal von Mises stress on the medial meniscus, and the maximal von Mises stress on the femoral cartilage in patients with knee osteoarthritis were significantly higher than those in the healthy control group. These biomechanical indicators, obtained through finite element analysis, quantitatively reflect the changes in the mechanical environment within the knee joint. The increased contact area in the study group may be related to the compensatory expansion of the load-bearing area after meniscus degeneration, while the increased maximum von Mises stress reflects the localized stress concentration, suggesting that the cartilage and meniscus undergo abnormal mechanical loading during the degenerative process.

In recent years, some studies (14, 23) have reported that patients with knee osteoarthritis exhibit alterations in knee joint kinematics. For instance, the range of knee flexion and extension during the early stance phase in these patients is lower than that in the normal population. This phenomenon may be attributed to an increased range of knee flexion, which shifts the ground reaction force to the posterior side of the knee earlier, thereby subjecting the knee joint to a greater load during movement. Consequently, this mechanism may help reduce joint load and alleviate joint pain. These findings are similar to those of this study. Currently, the analysis of knee joint biomechanical behavior largely relies on finite element analysis, a method that allows for non-invasive understanding of individual knee joint biomechanical changes. Hutchison et al. (24) have found through gait analysis that knee adduction moment, varus thrust, and peak knee flexion moment are closely associated with the severity of knee osteoarthritis and positively correlated with the pain severity of patients. Patients with knee osteoarthritis exhibit more pronounced meniscal dislocation, femoral cartilage defects, and medial joint space narrowing compared to normal individuals, resulting in a larger total knee contact area and higher femoral cartilage contact pressure and peak stress (25). A further analysis reveals that patients with knee osteoarthritis experience a reduced circumferential load-bearing capacity of the medial meniscus, leading to medial sliding of the femur during axial load application; consequently, the contact area between the lateral meniscus and cartilage decreases, resulting in an increased peak stress (26). The aforementioned process accelerates cartilage damage. When cartilage defects reach a critical point, excessive stress reduces the activity of cartilage cells in the damaged area, and this stress redistributes from high-load to low-load regions, causing further harm to the surrounding residual cartilage cells, ultimately leading to disease progression (27).

Finally, this study preliminarily demonstrated the clinical value of applying quantitative MRI indicators and biomechanical indicators in the diagnosis of knee osteoarthritis. The findings suggested that these indicators had good diagnostic efficiency. However, when patients were subdivided based on disease severity and subjected to further ROC analysis, only the maximal von Mises stress on the medial meniscus exhibited diagnostic value in patients with varying severities of knee osteoarthritis. The clinical significance of this finding should be interpreted with caution. The medial meniscus plays an important role in load transmission and stress distribution during knee weight-bearing. Changes in its maximum von Mises stress sensitively reflect changes in the biomechanical environment of the knee joint. From a pathophysiological perspective, as osteoarthritis progresses, degeneration and dysfunction of the medial meniscus lead to localized stress concentration. This may explain why the maximal von Mises stress on the medial meniscus can distinguish different degrees of disease severity. However, it should be acknowledged that biomechanical assessment based on finite element analysis is not yet widespread in routine clinical practice. This is mainly due to the technical complexity, time cost, and professional requirements of three-dimensional reconstruction of MRI images and finite element modeling. The clinical translational value of this study lies in providing a theoretical basis for the future development of simplified biomechanical assessment tools. The results suggest that the maximal von Mises stress on the medial meniscus may be a biomechanical indicator worthy of further investigation.

Regarding the clinical value of combined application of WORMS and biomechanical indicators, this study suggests that the two approaches may be complementary. WORMS scores reflect structural changes in the knee joint and are a morphological assessment indicator. Biomechanical indicators reflect functional changes in the knee joint and reveal the mechanical consequences caused by structural changes. The combined application of these two methods is conducive to understanding the pathological characteristics of knee osteoarthritis from both structural and functional dimensions, thereby providing a more comprehensive basis for the development of individualized treatment regimens. For example, patients with high WORMS but relatively normal biomechanical parameters may indicate good joint compensatory function. In contrast, patients with moderate WORMS but significantly abnormal biomechanical parameters require attention to joint mechanical imbalance, and orthopedic or offloading treatments should be considered.

The limitations of this study include: (1) the relatively small sample size, the single-center retrospective design, the absence of an independent validation cohort, and the practical difficulties in the clinical application of the finite element analysis; (2) the lack of multivariable analysis to construct a combined diagnostic model; (3) the relatively limited sample size (60 patients and 50 controls). Although the ROC analysis demonstrated acceptable 95% confidence intervals for each indicator, future multicenter studies with larger samples are still needed to improve the extrapolation and robustness of the results. Large-scale, multicenter prospective studies are needed to explore simplified biomechanical assessment methods, integrate MRI with biomechanical parameters, and construct visualization tools such as nomograms, thereby improving clinical application value.

MRI indicators and biomechanical indicators in patients with knee osteoarthritis show significant differences compared to healthy individuals. The maximal von Mises stress on the femoral cartilage shifts to the medial border, and the maximal von Mises stress on the medial meniscus moves towards the medial border of the meniscus. These changes further cause intra-articular biomechanical disorders. The application of WORMS and biomechanical indicators in the diagnosis of knee osteoarthritis has a promising prospect.

## Data Availability

The original contributions presented in the study are included in the article/Supplementary Material, further inquiries can be directed to the corresponding authors.

## References

[1] Katz JN Arant KR Loeser RF. Diagnosis and treatment of hip and knee osteoarthritis: a review. Jama. (2021) 325:568–78. 10.1001/jama.2020.22171 33560326 PMC8225295

[2] Li J Fu S Gong Z Zhu Z Zeng D Cao P MRI-based texture analysis of infrapatellar fat pad to predict knee osteoarthritis incidence. Radiology. (2022) 304:611–21. 10.1148/radiol.212009 35638929 PMC9434820

[3] Sukerkar PA Doyle Z. Imaging of osteoarthritis of the knee. Radiol Clin North Am. (2022) 60:605–16. 10.1016/j.rcl.2022.03.004 35672093

[4] Chaudhari AS Kogan F Pedoia V Majumdar S Gold GE Hargreaves BA. Rapid knee MRI acquisition and analysis techniques for imaging osteoarthritis. J Magn Reson Imaging. (2020) 52:1321–39. 10.1002/jmri.26991 31755191 PMC7925938

[5] Mahmoudian A Lohmander LS Mobasheri A Englund M Luyten FP. Early-stage symptomatic osteoarthritis of the knee—time for action. Nat Rev Rheumatol. (2021) 17:621–32. 10.1038/s41584-021-00673-4 34465902

[6] Dainese P Wyngaert KV De Mits S Wittoek R Van Ginckel A Calders P. Association between knee inflammation and knee pain in patients with knee osteoarthritis: a systematic review. Osteoarthritis Cartilage. (2022) 30:516–34. 10.1016/j.joca.2021.12.003 34968719

[7] Lee WS Kim HJ Kim KI Kim GB Jin W. Intra-articular injection of autologous adipose tissue-derived mesenchymal stem cells for the treatment of knee osteoarthritis: a phase IIb, randomized, placebo-controlled clinical trial. Stem Cells Transl Med. (2019) 8:504–11. 10.1002/sctm.18-0122 30835956 PMC6525553

[8] Lombardi AF Ma Y Jang H Jerban S Tang Q Searleman AC AcidoCEST-UTE MRI reveals an acidic microenvironment in knee osteoarthritis. Int J Mol Sci. (2022) 23:4466. 10.3390/ijms23084466 35457284 PMC9027981

[9] Jansen NEJ Molendijk E Schiphof D van Meurs JBJ Oei EHG van Middelkoop M Metabolic syndrome and the progression of knee osteoarthritis on MRI. Osteoarthritis Cartilage. (2023) 31:647–55. 10.1016/j.joca.2023.02.003 36801367

[10] Shakoor D Demehri S Roemer FW Loeuille D Felson DT Guermazi A. Are contrast-enhanced and non-contrast MRI findings reflecting synovial inflammation in knee osteoarthritis: a meta-analysis of observational studies. Osteoarthritis Cartilage. (2020) 28:126–36. 10.1016/j.joca.2019.10.008 31678664

[11] Ghouri A Muzumdar S Barr AJ Robinson E Murdoch C Kingsbury SR The relationship between meniscal pathologies, cartilage loss, joint replacement and pain in knee osteoarthritis: a systematic review. Osteoarthritis Cartilage. (2022) 30:1287–327. 10.1016/j.joca.2022.08.002 35963512

[12] Culvenor AG Øiestad BE Hart HF Stefanik JJ Guermazi A Crossley KM. Prevalence of knee osteoarthritis features on magnetic resonance imaging in asymptomatic uninjured adults: a systematic review and meta-analysis. Br J Sports Med. (2019) 53:1268–78. 10.1136/bjsports-2018-099257 29886437 PMC6837253

[13] Weizel A Distler T Detsch R Boccaccini AR Bräuer L Paulsen F Hyperelastic parameter identification of human articular cartilage and substitute materials. J Mech Behav Biomed Mater. (2022) 133:105292. 10.1016/j.jmbbm.2022.105292 35689988

[14] Khoury MA Chamari K Tabben M Alkhelaifi K Papacostas E Marín Fermín T Knee osteoarthritis: clinical and MRI outcomes after multiple intra-articular injections with expanded autologous adipose-derived stromal cells or platelet-rich plasma. Cartilage. (2023) 14:433–44. 10.1177/19476035231166127 37350015 PMC10807730

[15] Murphy AN Yelverton B Clesham K Hassell K Kavanagh E Eustace S Does MRI knee in those over 50 years with knee pain in osteoarthritis Alter management? a retrospective review. J Knee Surg. (2023) 36:584–90. 10.1055/s-0041-1740390 34879407

[16] Wang Z Jones G Winzenberg T Cai G Laslett LL Aitken D Effectiveness of curcuma longa extract for the treatment of symptoms and effusion-synovitis of knee osteoarthritis: a randomized trial. Ann Intern Med. (2020) 173:861–9. 10.7326/m20-0990 32926799

[17] Wang Z Winzenberg T Singh A Aitken D Blizzard L Boesen M Effect of curcuma longa extract on serum inflammatory markers and MRI-based synovitis in knee osteoarthritis: secondary analyses from the curkoa randomised trial. Phytomedicine. (2023) 109:154616. 10.1016/j.phymed.2022.154616 36610110

[18] Richard MJ Driban JB McAlindon TE. Pharmaceutical treatment of osteoarthritis. Osteoarthritis Cartilage. (2023) 31:458–66. 10.1016/j.joca.2022.11.005 36414224

[19] Pishgar F Guermazi A Roemer FW Link TM Demehri S. Conventional MRI-based subchondral trabecular biomarkers as predictors of knee osteoarthritis progression: data from the osteoarthritis initiative. Eur Radiol. (2021) 31:3564–73. 10.1007/s00330-020-07512-2 33241511 PMC9583892

[20] Luo P Hu W Jiang L Chang S Wu D Li G Evaluation of articular cartilage in knee osteoarthritis using hybrid multidimensional MRI. Clin Radiol. (2022) 77:e518–25. 10.1016/j.crad.2022.03.002 35469665

[21] Pishgar F Ashraf-Ganjouei A Dolatshahi M Guermazi A Zikria B Cao X Conventional MRI-derived subchondral trabecular biomarkers and their association with knee cartilage volume loss as early as 1 year: a longitudinal analysis from osteoarthritis initiative. Skeletal Radiol. (2022) 51:1959–66. 10.1007/s00256-022-04042-4 35366094 PMC9414671

[22] Gu YG Jiang H. Correlation between synovitis and traditional Chinese medicine syndromes of knee osteoarthritis in WORMS score. Zhongguo Gu Shang. (2019) 32:1108–11. 10.3969/j.issn.1003-0034.2019.12.008 31870068

[23] Aljehani MS Christensen JC Snyder-Mackler L Crenshaw J Brown A Zeni JA Jr. Knee biomechanics and contralateral knee osteoarthritis progression after total knee arthroplasty. Gait Posture. (2022) 91:266–75. 10.1016/j.gaitpost.2021.10.020 34775230 PMC8963526

[24] Hutchison L Grayson J Hiller C D’Souza N Kobayashi S Simic M. Relationship between knee biomechanics and pain in people with knee osteoarthritis: a systematic review and meta-analysis. Arthritis Care Res (Hoboken). (2023) 75:1351–61. 10.1002/acr.25001 35997473

[25] Pinto RF Birmingham TB Philpott HT Primeau CA Leitch KM Arsenault DA Changes and associations between gait biomechanics and knee inflammation after aspiration and glucocorticoid injection for knee osteoarthritis. Arthritis Care Res (Hoboken). (2023) 75:1764–72. 10.1002/acr.25064 36478406

[26] Schrijvers JC Rutherford D Richards R van den Noort JC van der Esch M Harlaar J. Inter-laboratory comparison of knee biomechanics and muscle activation patterns during gait in patients with knee osteoarthritis. Knee. (2021) 29:500–9. 10.1016/j.knee.2021.03.001 33756260

[27] Young-Shand KL Roy PC Dunbar MJ Abidi SSR Astephen Wilson JL. Gait biomechanics phenotypes among total knee arthroplasty candidates by machine learning cluster analysis. J Orthop Res. (2023) 41:335–44. 10.1002/jor.25363 35538599

